# Implications of Dengue Outbreaks for Blood Supply, Australia

**DOI:** 10.3201/eid1905.121664

**Published:** 2013-05

**Authors:** Helen M. Faddy, Clive R. Seed, Jesse J. Fryk, Catherine A. Hyland, Scott A. Ritchie, Carmel T. Taylor, Kathryn L. Van Der Merwe, Robert L.P. Flower, William J.H. McBride

**Affiliations:** Australian Red Cross Blood Service, Kelvin Grove, Queensland, Australia (H.M. Faddy, J.J. Fryk, C.A. Hyland, K.L. Van Der Merwe, R.L.P. Flower);; Australian Red Cross Blood Service, Osborne Park, Western Australia, Australia (C.R. Seed);; James Cook University, Cairns, Queensland, Australia (W.J.H. McBride, S.A. Ritchie);; Queensland Health, Cairns (S.A. Ritchie);; Queensland Health Forensic and Scientific Services, Coopers Plains, Queensland, Australia (C.T. Taylor)

**Keywords:** Dengue virus, Australia, blood donor, seroprevalence, asymptomatic, risk, cost, vector-borne infections, viruses

## Abstract

Dengue outbreaks have increased in size and frequency in Australia, and transfusion-transmitted dengue poses a risk to transfusion safety. Using whole blood samples collected during the large 2008–2009 dengue epidemic, we estimated the risk for a dengue-infectious blood donation as ≈1 in 7,146 (range 2,218–50,021).

Dengue causes >50 million infections per year worldwide; however, the true incidence is expected to be higher given that asymptomatic infection is possible (*1*). Dengue virus types 1–4 (DENV-1–4) are emerging or reemerging in many regions of the world (*1*,*2*), including Australia (*3*). One of the largest epidemics in at least 50 years occurred in Queensland, Australia, during 2008–2009, with separate outbreaks in Cairns (and surrounding regions; DENV-2, DENV-3; 2008–2009), Innisfail (DENV-4; 2009), and Townsville (DENV-1, DENV-3; 2009), totaling >1,000 confirmed clinical cases (*3*).

Infection with DENVs poses a risk for transfusion safety, and 5 cases of transfusion-transmitted dengue have been reported (*4*,*5*). In addition, DENV RNA has been detected in asymptomatic blood donors from areas to which dengue is endemic (*6*–*8*). Given the absence of an approved blood screening test for dengue in Australia, managing transfusion-transmission risk focuses on identifying donors at risk for exposure and temporarily excluding them from donating fresh blood components (erythrocytes, platelets, and clinical plasma) (referred to here as dengue management strategy) (*9*). Plasma collection for fractionation can continue because the process of manufacturing concentrates inactivates the virus (*10*). This approach assists with meeting an expanding demand for intravenous Ig but may result in fresh component losses and be associated with considerable cost.

Risk to the blood supply correlates with asymptomatic donor viremia; understanding the rate of dengue subclinical infection in countries to which it is not endemic and local northern Queensland seroprevalence is necessary for assessing this risk. We examined dengue seroprevalence rates in Australian donors during this epidemic; used these data to estimate the subclinical infection rate, population prevalence, and associated transfusion-transmission risk; and estimated the economic effect of this epidemic to the Australian Red Cross Blood Service (Blood Service).

## The Study

Whole blood samples collected during the 2008–2009 dengue epidemic were tested for DENV IgM by ELISA (Dengue IgM Capture ELISA; Panbio, Brisbane, QLD, Australia). All reactive samples were tested with a second ELISA (Anti-Dengue IgM ELISA; Standard Diagnostics Inc., Giheung-gu, South Korea) and by the Public Health Virology Laboratory at Queensland Health Forensic and Scientific Services (QHFSS) (*11*). Serologic evidence of recent exposure (presence of DENV IgM) was observed in 12 (0.22%) donors (Table 1). Of the 8 DENV IgM–positive samples that were examined for type specificity, 7 (88%) were DENV-3 specific, which was the dominant type during the epidemic (*3*).

**Table 1:** Serologic evidence of recent dengue exposure by dengue virus IgM in blood donors, northern Queensland, Australia, 2008–2009 epidemic

Location	Donors			Donations	
	No. samples	Reactive samples, no. (% [95% CI])		No. samples	Reactive samples, no. (% [95% CI])
North Queensland	5,453	12 (0.22 [0.10–0.34])		10,026	13 (0.13 [0.06–0.20])
Cairns	2,416	8 (0.33 [0.10–0.56])		5,051	9 (0.18 [0.06–0.29])
Townsville	3,037	4 (0.13 [0.00–0.26])		4,975	4 (0.08 [0.00–0.16])

We used these IgM seroprevalence rates to estimate the rate of subclinical dengue infection (Technical Appendix). We estimated 168–921 subclinical cases (clinical:subclinical ratio 1.0:0.59; range 0.18–1.0) in Cairns, the city where the epidemic was centered. Our estimate was toward the lower end of that observed in dengue-endemic areas (*12*,*13*) but higher than that estimated during a DENV-2 outbreak in Charters Towers (*14*), which probably reflects the different methods used in the respective studies.

Selected samples were tested for DENV IgG by ELISA (Dengue IgG Indirect ELISA; Panbio). All reactive samples were tested at QHFSS. Serologic evidence of previous exposure (presence of DENV IgG) was influenced overall by donor location (p<0.05) and age (p<0.05). The proportion of the northern Queensland donor population with DENV IgG was 9.43% (95% CI 7.98%–10.89%), and this proportion increased with age (Table 2), which indicates cumulative previous exposure. The proportion of Melbourne (control area with no dengue activity) donors with DENV IgG was 6.78% (95% CI 4.48%–9.09%); however, no change was observed with age (Table 2), demonstrating no cumulative exposure. Previous exposure in Melbourne was surprisingly high; these persons may have been exposed during travel to dengue-endemic areas (subsequent follow-up demonstrated 94% reported travel to countries to which dengue may be endemic).

**Table 2:** Prevalence of dengue virus IgG in the blood donor population, northern Queensland, Australia

Variable	No. samples	Reactive, no. (% [95% CI])
Northern Queensland	1,548	146 (9.43 [7.98–10.89])
Age group, y		
<40	567	18 (3.17 [1.73–4.62])
40–60	742	70 (9.43 [7.33–11.54])
>60	239	58 (24.27 [18.83–29.70])
Outbreak		
Start	788	72 (9.14 [7.13–11.15])
End	760	74 (9.74 [7.63–11.84])
Cairns	738	53 (7.18 [5.32–9.04])
Age group, y		
<40	271	5 (1.85 [0.24–3.45])
40–60	360	26 (7.22 [4.55–9.90])
>60	107	22 (20.56 [12.90–28.22])
Outbreak		
Start	384	31 (8.07 [5.35–10.80])
End	354	22 (6.21 [3.70–8.73])
Townsville	810	93 (11.48 [9.29–13.68])
Age group, y		
<40	296	13 (4.39 [2.06–6.73])
40–60	382	44 (11.52 [8.32–14.72])
>60	132	36 (27.27 [19.68–34.87])
Outbreak		
Start	404	41 (10.15 [7.20–13.09])
End	406	52 (12.81 [9.5–16.066])
Melbourne	457	31 (6.78 [4.48–9.09])
Age group, y		
<40	151	11 (7.28 [3.14–11.43])
40–60	215	16 (7.44 [3.93–10.95])
>60	91	4 (4.40 [0.18–8.61])

The proportion of donors with DENV IgG did not change from the beginning to the end of the outbreaks in Cairns and Townsville, nor in northern Queensland as a whole (Table 2), which suggests that the epidemic was not of a scale to result in a change in population seroprevalence. This study was powered to detect a change in incidence of at least 10%; small changes might have been missed, which would be difficult to detect through such studies.

We used our seroprevalence data along with donation frequencies to estimate the risk of collecting a dengue-infectious donation, based on published models (*9*,*15*) (Technical Appendix). Using this model, the risk of collecting a dengue-infectious donation in Cairns during the epidemic was ≈1 in 7,146 (range 2,218–50,021) (Figure). These estimates are similar to those obtained by using a published probabilistic model (*9*) revised to incorporate the outbreak specific subclinical infection rate reported herein, which predicts the risk to be ≈1 in 9,303 (range 3,092–32,344) donations in Cairns. Because both methods derive estimates within comparable ranges, it would appear valid to use the revised probabilistic model as a predictive risk estimator during future outbreaks.

**Figure:**
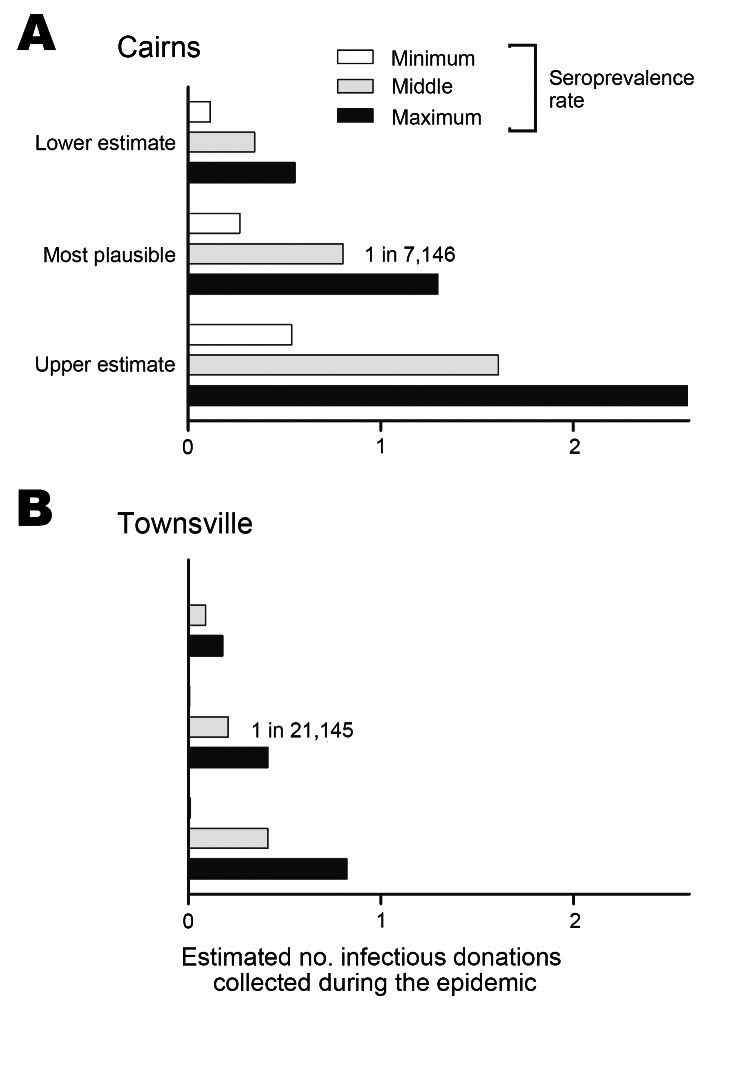
Risk of collecting a dengue-infectious blood donation, northern Queensland, Australia, 2008–2009 epidemic. Estimated risk calculated for Cairns (A) and Townsville (B).

The dengue management strategy used during the epidemic cost the Blood Service ≈1–3.8 million Australian dollars (2009 terms). This estimate is publically available and was based on: the number of donations affected by the dengue management strategy, collection targets for 2009, costs associated with whole blood collections, additional costs to meet national targets, transportation costs to meet demand in affected regions, and additional waste costs. An offset for any plasma obtained through a whole blood donation (used for fractionation) was included in selected estimates.

## Conclusions

Subclinical dengue infection rates vary by population, specific outbreak, and area examined (*12*,*14*). We demonstrate that the clinical to subclinical infection rate during the 2008–2009 dengue epidemic in northern Queensland, where dengue occurs seasonally, was toward the lower end of that observed in dengue-endemic countries (*12*,*13*). This observation, together with our data suggesting that the incidence of dengue in the northern Queensland population did not change from the beginning to the end of the epidemic, suggests the control and clinical management of dengue during this epidemic was comprehensive.

We also estimated that the risk of collecting a dengue-infectious blood donation in Cairns during the epidemic was ≈1 in 7,146 (range 2,218–50,021). Given these risks, the increasing need for plasma in Australia, and the absence of a screening test for blood donations in Australia, the continuation of the dengue management strategy during future outbreaks is warranted. However, this strategy may have added strain on the inventory available to meet clinical demand for fresh blood components and was associated with a cost to the Blood Service of >1 million Australian dollars. Although this strategy is a necessary precaution to maintain safety, alternative approaches may exist, such as implementation of a suitable screening test (were one available) or pathogen reduction technology (a process designed to inactivate pathogens in blood products), which may offer a similar level of safety but be more cost effective. With dengue becoming increasingly common in Australia (*3*) and the world (*1*), these alternative approaches may be needed in the future.

Technical Appendix Use of IgM seroprevalence rates to estimate the rate of subclinical dengue virus infection and associated transfusion-transmission risk.
