# Supplementary material for: Implications of Dengue Outbreaks for Blood Supply, Australia
Source: Emerg Infect Dis. 2013 May;19(5):787–9. doi: 10.3201/eid1905.121664 (PMC3647514; doi:10.3201/eid1905.121664)
Supplement: Technical Appendix — Use of IgM seroprevalence rates to estimate the rate of subclinical dengue virus infection and associated transfusion-transmission risk. [file 12-1664-Techapp-s1.pdf]

Article DOI: http://dx.doi.org/10.3201/eid1905.121664

Implications of Dengue Outbreaks for Blood Supply, Australia

Technical Appendix

Statistical Analysis

Sample numbers for dengue virus (DENV) IgG testing were determined by using a power analysis (*1*) to enable detection of a 10% difference in prevalence between the beginning and end of the Cairns and Townsville outbreaks with at least 85% power. The proportion of donations/donors with the presence of antibody was determined and a 95% CI calculated. Logistic regression modeling was used to assess the relationship of donor sex, age, blood group (ABO and Rh antigens), and location with DENV IgG status. The antibody response (reactive or nonreactive) was the dependent variable, with sex, location, blood group, and age group as factors. The goodness-of-fit of the model was assessed by using the Deviance and Pearson χ^2 tests. The Statistical Package for the Social Sciences (SPSS; IMB Australia Ltd., St. Leonards, NSW, Australia) was used for analyses. Significance was determined with a p value $\leq$0.05.

Subclinical Infection Estimate

Given the length of DENV IgM persistence (6 months for primary dengue infection) (*2*) and the timing of the last reported locally acquired dengue case in North Queensland before this epidemic (early 2008) (*3*), it is likely that all donors donating during the 2008–2009 dengue epidemic and who were positive for DENV IgM were exposed during this epidemic. To be eligible to donate blood in Australia, donors must satisfy a number of criteria, including being well at the time of the donation. It is therefore highly unlikely that a donor would have been eligible to donate if he or she had a symptomatic dengue infection. Furthermore, donors are requested to inform the Blood Service if they experience any illness within 7 days of donation so their donation can be quarantined or recalled, ruling out donors with symptomatic infection who may have donated during the presymptomatic viremic period. Assuming 100% compliance with Blood Service policy, that any donor with DENV IgM was exposed during the 2008–2009 epidemic and that the blood donor population had a

similar level of dengue exposure as the general population, we utilized IgM seroprevalence rates, along with estimates of the Cairns (164,354) and Townsville (181,740) populations in 2009 (*4*), to estimate the rate of subclinical dengue infection. Although seroprevalence data without detailed clinical information will not produce a definitive answer here, it will provide an estimate of the scale of the problem, independent of current notification systems. These estimates of subclinical infection do not include ill “non-reporters” who did not seek medical attention and who were unable to donate, so although our estimate is realistic, it is likely to underestimate the true number of infections during the epidemic. Moreover, our estimate assumes that all dengue cases during the epidemic were actually notified; the subclinical rate could of course be lower depending on the number of cases that were not notified as a ratio of those that were.

Example Calculation (Cairns)

NOTE: All calculations were performed by using Microsoft Excel (Redmond, WA, USA). As such, rounding may have led to slight variation when compared with manual calculations.

Population = 164,354 (*4*)

DENV IgM seroprevalence rate (Cairns) = 0.33% (95% CI 0.10–0.56%) (Table 1)

Estimated number of subclinical cases (lower limit) = DENV IgM seroprevalence rate (lower 95% CI) × Population (or 0.10% × 164,354 = 168)

Estimated number of subclinical cases (most probable) = DENV IgM seroprevalence rate × Population (or 0.33% × 164,354 = 544)

Estimated number of subclinical cases (upper limit) = DENV IgM seroprevalence rate (upper 95% CI) × Population (or 0.56% × 164,354 = 921)

Therefore, we estimate that there were between 168 and 921 subclinical cases in Cairns during the epidemic.

The number of clinical cases in Cairns during the epidemic was 917 (*3*). Using the subclinical estimates described above, we estimate a clinical to subclinical ratio of 1.0:0.59 ((1/917) × 544) with a range of 1.0:0.18 ([1/917]) × 168) to 1.0:1.0 ([1/917] × 921).

Risk Analyses

Two different models were used to estimate the transfusion-transmission risk during the 2008–2009 dengue epidemic. The first estimates the risk of collecting a dengue-infectious donation based on our seroprevalence data from blood donations (Table 1) along with the donor donation frequency for each city during the outbreak period (which included 3 months after the last confirmed case) and is based on published models (*5,6*). Theoretically there are 3 sources of obtaining a viremic donation: 1) asymptomatic cases; 2) clinical cases that may slip through the screening process; and 3) donors who may donate during the 1–2 day presymptomatic viremic period (*7*). As mentioned earlier, the Blood Service has policies in place to ensure the latter 2 scenarios are minimized, so this risk model is based on the first source only.

The probability of collecting a dengue-infectious donation was estimated for the duration of the Cairns and Townsville outbreaks separately. We estimate the probability of collecting an infectious donation ($P_{\text{infectious donation}}$) as

$P_{\text{infectious donation}}$ = (length of viremia in days for asymptomatic infection/length of IgM persistence in days [plus the IgM-negative viremic period]) × IgM donation seroprevalence during the outbreak × donor donation frequency during the outbreak.

This probability was then used to predict the number of infectious blood donations ($N_{\text{infectious donations}}$) collected over the course of the outbreak by

$N_{\text{infectiousc donations}}$ = $P_{\text{infectious donation}}$ × number of donations collected during the outbreak.

The duration of viremia was derived from a range of published studies, and assuming the duration of viremia in asymptomatic infection is similar to what is observed in clinical cases (*6,8–10*), we have estimated that it ranges from 3 to 14 days (most plausible estimate 7 days). The duration of IgM seropositivity was based on 6 months of persistence (*2*). Given that a period of viremia exists before the development of an IgM response, approximated as 5 days for the purposes of this analysis, it was necessary to adjust for this by adding this period of time to the duration of IgM seropositivity. The IgM seroprevalence rate obtained for donations collected during the outbreak for each region was modeled (middle estimate) along with the upper (maximum estimate) and lower (minimum estimate) 95% CI for each proportion. In addition, the number of donations, which had the potential to result in a blood component, collected during the epidemic was retrospectively obtained.

Example Calculation (Cairns, most plausible estimate [length of asymptomatic viremia], medium estimate for IgM seroprevelance)

NOTE: All calculations were performed by using Microsoft Excel. As such, rounding may have led to slight variation when compared with manual calculations.

Length of viremia in days for asymptomatic infection = 7 days

Length of IgM persistence in days (plus the IgM negative viremic period) = 187 (6 months + 5 days)

IgM donation seroprevalence during the outbreak (Cairns) = 0.18%

Number of donations collected during the outbreak (Cairns) = 5,753

Number of donors giving successful donations during the outbreak (Cairns) = 2,770

Donor donation frequency during the outbreak = 2.0769

$P_{\text{infectious donation}}$ = (7/187) × 0.18% × 2.0769 = 0.000139941

$N_{\text{infectiousc donations}}$ = 0.000139941 × 5,753 = 0.805077923

$Risk_{\text{collecting an infectious unit}}$ = (1/0.805077923) × 5,753 = 7,146 (or 1 in every 7,146 donations)

A published probabilistic model was also used, which estimates the risk for dengue transmission by blood using the number of confirmed cases during the epidemic (also including 3 months after the last confirmed case) (*6*). This model was adapted to incorporate the subclinical infection rate (including the upper and lower 95% CI) estimated from the seroprevalence data for these outbreaks from this study, which addresses 1 key limitation of the original model. The specifics of this model are described elsewhere (*6*). In this study, we applied this model to the 2008–2009 dengue outbreaks in Cairns and Townsville.

References

1. Moore DS, McCabe GP. Introduction to the practice of statistics. 3rd ed. New York: WH Freeman and Company; 1999.

2. Prince HE, Matud JL. Estimation of dengue virus IgM persistence using regression analysis. Clin Vaccine Immunol. 2011;18:2183–5. PubMed http://dx.doi.org/10.1128/CVI.05425-11

3. Fitzsimmons GJ, Wright P, Johansen CA, Whelan PI. Arboviral diseases and malaria in Australia, 200809: annual report of the National Arbovirus and Malaria Advisory Committee. Commun Dis Intell. 2010;34:225–40.

4. Australian Bureau of Statistics. 3218.0 Regional population growth, Australia as of 30 March 2012 [cited 2012 May 14]. http://www.abs.gov.au/AUSSTATS/abs@.nsf/DetailsPage/3218.02010-11

5. Weinstein P, Weinstein SR, Rowe RJ. Transfusions: how many cases of Ross River virus infection do we cause? Med J Aust. 1995;163:276. PubMed

6. Seed CR, Kiely P, Hyland CA, Keller AJ. The risk of dengue transmission by blood during a 2004 outbreak in Cairns, Australia. Transfusion. 2009;49:1482–7. PubMed http://dx.doi.org/10.1111/j.1537-2995.2009.02159.x

7. Guzmán MG, Kouri G. Dengue: an update. Lancet Infect Dis. 2002;2:33–42. PubMed http://dx.doi.org/10.1016/S1473-3099(01)00171-2

8. Vaughn DW, Green S, Kalayanarooj S, Innis BL, Nimmannitya S, Suntayakorn S, et al. Dengue viremia titer, antibody response pattern, and virus serotype correlate with disease severity. J Infect Dis. 2000;181:2–9. PubMed http://dx.doi.org/10.1086/315215

9. Gubler DJ. Dengue and dengue hemorrhagic fever. Clin Microbiol Rev. 1998;11:480–96. PubMed

10. Murgue B, Roche C, Chungue E, Deparis X. Prospective study of the duration and magnitude of viraemia in children hospitalised during the 1996-1997 dengue-2 outbreak in French Polynesia. J Med Virol. 2000;60:432–8. PubMed http://dx.doi.org/10.1002/(SICI)1096-9071(200004)60:4<432::AID-JMV11>3.0.CO;2-7
